# The scaffolding protein IQGAP1 enhances EGFR signaling by promoting oligomerization and preventing degradation

**DOI:** 10.1016/j.jbc.2024.107844

**Published:** 2024-09-30

**Authors:** V. Siddartha Yerramilli, Guanyu Lin, Jessica L. Reisinger, Rachel M. Hemmerlin, Samantha K. Lindberg, Karin Plante, Alonzo H. Ross, Arne Gericke, Suzanne Scarlata

**Affiliations:** 1 Department of Chemistry and Biochemistry, Worcester Polytechnic Institute, Worcester, Massachusetts, USA; 2 Department of Biochemistry and Molecular Pharmacology, University of Massachusetts Medical School, Worcester, Massachusetts, USA

**Keywords:** IQGAP, EGFR, fluorescence imaging, protein oligomerization, cell signaling

## Abstract

IQGAP1 is a large, multi-domain scaffold that connects and modulates different signaling networks including the one initiated by epidermal growth factor (EGF). In this study, we have used live cell fluorescence imaging methods along with other biochemical techniques to follow the mechanisms used by IQGAP1 to enhance EGF signaling. We show that IQGAP1 enhances EGF signaling by promoting the oligomerization of its receptor, EGFR, upon EGF addition along with concurrent IQGAP oligomerization. Using cellular markers, we find that IQGAP1 promotes the plasma membrane localization of EGFR and promotes association to one of its phosphoinositide lipid pathway ligands, PI(3,4,5)P_3_. Additionally, we find that binding of EGFR to IQGAP1 protects EGFR from lysosomal degradation. Taken together, our results show that IQGAP1 enhances EGF-mediated pathway progression through mechanisms that augment simple scaffolding activities.

IQGAP1 (IQ-domain containing Ras GTPase Activating Protein 1) is a multidomain protein involved in mediating multiple cell activities. IQGAP1 lacks catalytic activity (1) but serves as a scaffold for components of several signaling pathways (2) such as the PI3K/Akt pathway, where IQGAP1 binds PI(4,5)P_2_ and PI(3,4,5)P_3_, the phosphokinases involved in their generation (*i.e.* PIPKI and PI3K), and downstream signaling molecules such as Akt and PDK1 (3, 4, 5). IQGAP1 is also thought to modulate the epidermal growth factor (EGR) signaling pathway that mediates growth (6). IQGAP1 is known to directly bind to EGFR and has been thought to modulate EGFR’s activation (7) although this idea has not been directly shown. However, it has been shown that EGFR binding to IQGAP1 specifically modulates the PI3K-Akt pathway (8).

EGFR is a member of the tyrosine kinase family (9) where ligand binding induces receptor dimerization (10) leading to phosphorylation and subsequent activation of downstream signals that include the PI3K/Akt and Ras/Erk pathways (6, 11). The signal from activated EGFR is terminated through endocytosis of ligated EGFR oligomers in early endosomes (12). Two major destinations exist for EGFR trafficking: return to the cell surface or lysosomal degradation. These pathways balance enhanced signaling from recycled EGFR at the cell surface against signal attenuation *via* the degradative pathway. Dysregulation of EGFR signaling results in gene amplification and overexpression, and activating mutations of EGFR have been identified in a variety of cancers (13). These mutations include ones that cause trafficking defects of EGFR (14). Because of its dysregulation in many cancers, EGFR has been used as a target with mixed success (15, 16) pointing to the need for alternative approaches.

Like EGFR, increased IQGAP1 expression has also been found in a variety of cancers (17, 18, 19, 20, 21, 22, 23). IQGAP1 plays a role in receptor trafficking through its interactions with several endosomal and lysosomal proteins, including the recycling endosomes-associated Rab11 (24). IQGAP1 is also involved in the trafficking and recycling of the chemokine receptor CXR4 (25), and forms complexes with PIPKI*γ* by way of endosomal transportation possibly by physically scaffolding endosomes and microtubules. IQGAP1 is also known to impact EGFR signaling. A mutant that blocks IQGAP1’s binding to phosphoinositide kinases was shown to suppress EGF-mediated activation of the PI3K-Akt but not the Ras-ERK pathway. This mutant did not result in changes in colocalization between IQGAP1 and internalized EGFR (8), suggesting that there may be multiple sites for IQGAP1 to interact with EGFR.

Here, we have studied the role of IQGAP1 in EGFR signaling, including changes in EGFR localization and oligomerization by fluorescence imaging. We find that IQGAP1 scaffolds EGFR to the pools of PI(3,4,5)P_3_, and protects EGFR against lysosomal degradation. Our studies show that IQGAP1 offers an additional level of EGFR regulation.

## Results

### IQGAP1 oligomerizes upon cell stimulation with EGF

**Figure 1:**
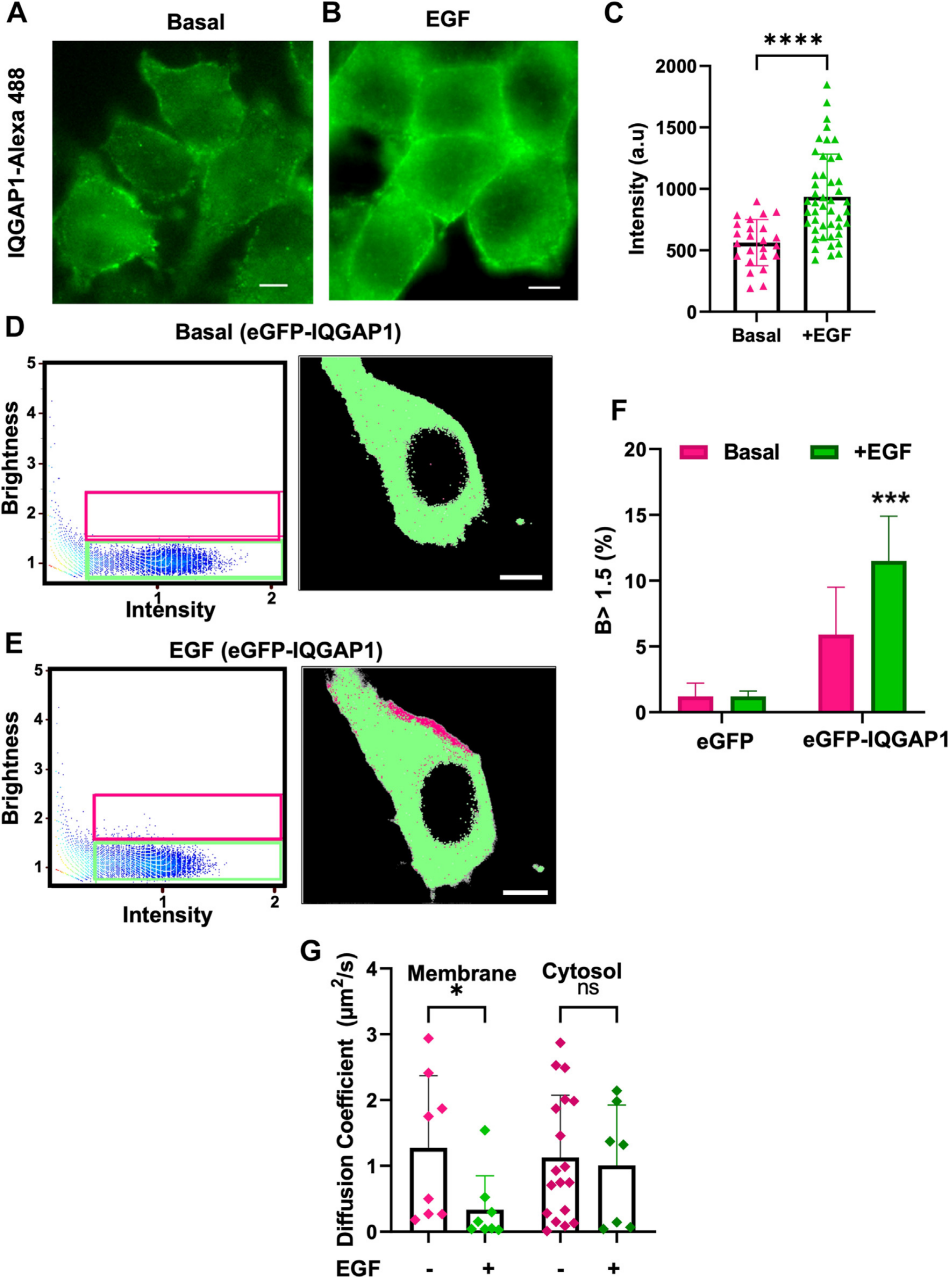
**IQGAP1 forms membrane complexes in response to EGF stimulation.***A*, a representative image showing endogenous IQGAP1 detected with Alexa 488 secondary antibody in HeLa cells. *B*, a representative image showing endogenous IQGAP1 antibody detected with Alexa 488 secondary antibody in HeLa cells stimulated with 100 ng/ml EGF. *C*, the fluorescence intensity of endogenous IQGAP1 detected with Alexa 488 is quantified manually along the plasma membrane on images of HeLa cells before and after stimulation with 100 ng/ml EGF. *D*, N&B results displayed in a brightness vs intensity plot, where individual pixels of the HeLa cell images expressing eGFP-IQGAP1 are highlighted either in a *green box* (*B* < 1.5) or a *magenta box* (*B* > 1.5) (*left panels*). The distribution of these highlighted pixels can be seen overlaid (*right*) on a representative unstimulated cell. *B* > 1.5 values indicate IQGAP1 oligomerization. *E*, a brightness vs intensity plot, where individual pixels of images of HeLa cell expressing eGFP-IQGAP1 and stimulated with EGF are highlighted either in a *green box* (*B* < 1.5) or a *magenta box* (*B* > 1.5) (*left*). The distribution of these highlighted pixels can be seen overlaid (*right panels*) on a representative cell. This EGF-stimulated cell shows localization of IQGAP1 in higher order species at the plasma membrane. *F*, increase in the number of pixels having higher *B* values (*magenta pixels*, *B* > 1. 5) that represent oligomers of eGFP-IQGAP1 after the cells are stimulated with EGF compared to basal levels. *G*, the overall diffusion coefficients of eGFP-IQGAP1 particles derived from FCS correlation curves from plasma membrane-localized focal areas as well as cytosolic-localized on cells both at a basal state and after EGF stimulation. For more details, see Experimental Methods. n ≥ 10 at least two individual experiments, ∗*p* ≤ 0.05, ∗∗∗*p* < 0.001, and ∗∗∗∗*p* < 0.0001. Error bars denote standard deviation. Scale bar = 10 μm.

IQGAP1 binds a broad array of proteins (26). In HeLa cells, endogenous IQGAP1 is detected both at the plasma membrane and in the cytosol (Fig. 1, *A* and *B*). We stimulated cells with 100 ng/ml of EGF which is considered physiological (27, 28), as well as a higher dose of 1000 ng/ml with which we are able to elicit a robust stimulation in a short duration. EGF stimulation causes a statistically significant increase in IQGAP1 intensity localized to the plasma membrane, including cell-cell junctions (Fig. 1*C*), without a corresponding increase in intensity across the cell (Fig. S1*A*). To better understand these dynamics, we expressed eGFP-IQGAP1 in HeLa cells noting that the fluorescent tag does not interfere with its functionality (29, 30). We find a plasma membrane-localized increase in eGFP-IQGAP intensity upon EGF stimulation without a corresponding overall increase in eGFP fluorescence intensity (Fig. S1, *B* and *C*) correlating with increased localization of eGFP-IQGAP to the plasma membrane.

IQGAP1 is known to oligomerize (29, 31) and we wondered whether the shift in localization with EGF signaling seen in Fig. 1*A* is accompanied by changes in oligomerization. We assessed these changes by a quantitative spectroscopy-based method called Number & Brightness (N&B, see Methods). This method follows changes in the intensity and movement of pixels in a time series of fluorescent images that are analyzed to assess whether the movement corresponds to a monomer, as calibrated using free eGFP, or a higher order species. If we assign the *B* values for free eGFP in cells as 1.0, oligomerization is calculated as the percentage of pixels that correspond to a brightness (*B*) value above 1.0 after background correction (for further details, see (32)) (Fig. 1, *D* and *E*). The pixels representing higher order oligomers (*i.e., B* > 1.0) values can selected and their localization in the cell image assessed. In our studies, we note that monomeric eGFP in HeLa cells has a significant spread in B values and thus we can safely assume that *B* values above 1.5 represent oligomers. Therefore N&B for the quantification of oligomerization and visualization of where this oligomerization occurs. Note that at the resolution of these measurements, a pixel cannot be equated to a single molecule.

We collected a time series of images of HeLa cells expressing eGFP-IQGAP and analyzed changes in the location and intensity of all the pixels in the images over time by N&B. We see a significant increase of oligomeric species (*i.e.*, those with *B* value above 1.5) from ∼6% to ∼12% upon treatment with EGF (Fig. 1*F*), whereas no changes are seen for HeLa cells expressing free eGFP (Fig. 1*F*). This increase in the percentage of higher B values and the corresponding images indicates that EGF stimulation increases oligomerization and the plasma-membrane localization of IQGAP1 (Fig. 1, *D* and *E*). We observe a similar result in liver cancer cell line HepG2A (Fig. S1*D*).

We used fluorescence correlation spectroscopy (FCS) to observe the changes in the mobility of eGFP-IQGAP1 as it responds to EGF stimulation. FCS quantifies the diffusion coefficient of a fluorescent protein which will decrease if the protein aggregates to a size that is at least 5-fold larger. We see a significant decrease in the diffusion coefficient in the plasma membrane population of IQGAP1 with stimulation that accompanies the clustering observed by the N&B analysis (Fig. 1*G*), and although the significance is low, the data are clustered in such a way that indicate a difference. However, there is no change in the mobility of the cytosolic population. Noting that measurements of the plasma membrane population may also include a cytosolic component and *vice versa*, these studies indicate that EGF signaling promotes the formation of domains enriched in IQGAP1 on or close to the plasma membrane.

### IQGAP1 mediates EGFR levels and oligomerization

**Figure 2:**
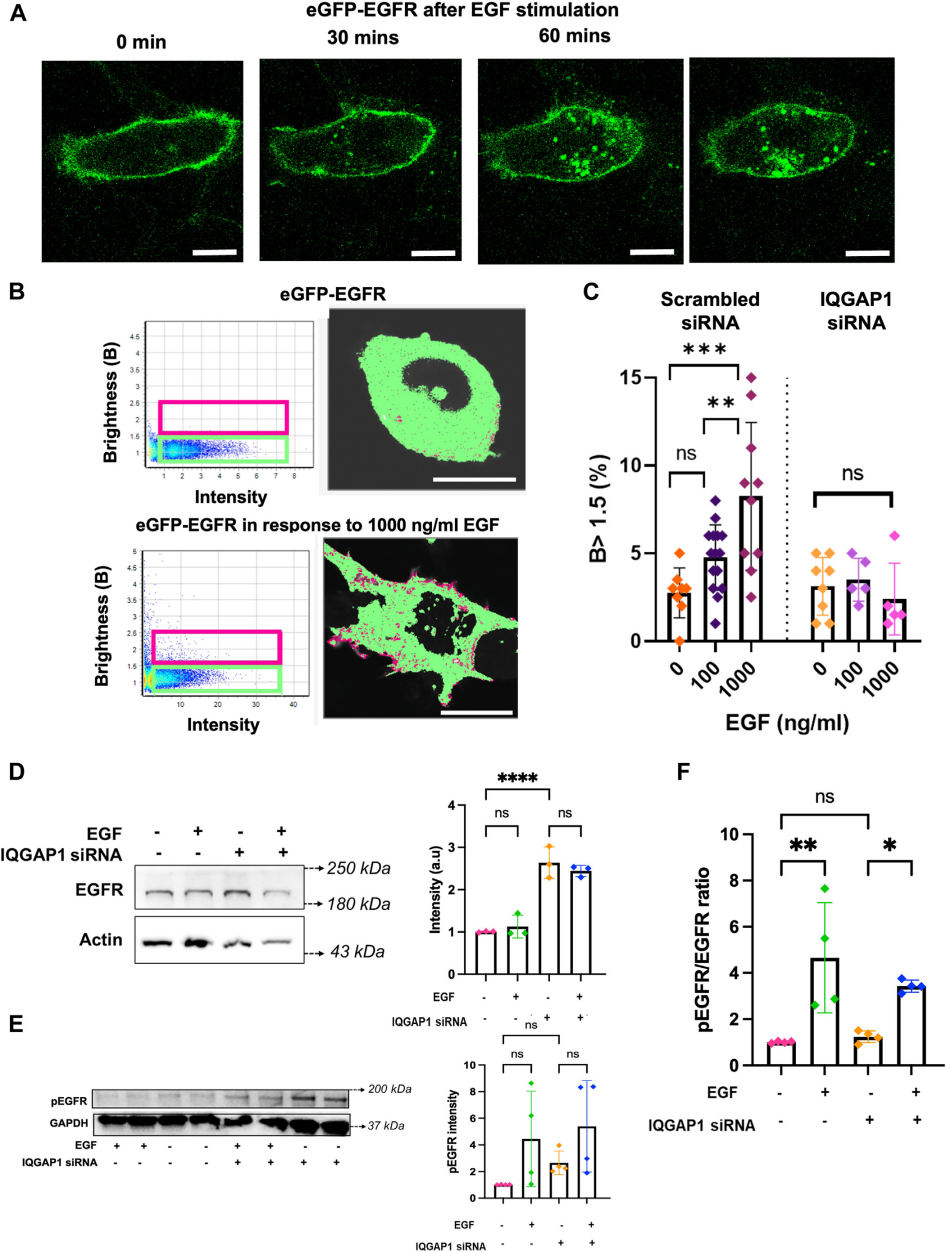
**IQGAP1 enhances EGFR localization to plasma membrane.***A*, images of HeLa cells expressing eGFP-EGFR taken immediately 30 min, 60 min and 90 min after incubation with EGF. *B*, N&B results plotted as a brightness vs intensity where individual pixels of the images of HeLa cells expressing eGFP-EGFR are highlighted either in a *green box* (*B* < 1.5) or a *magenta box* (*B* > 1.5) (*left*). The distribution of these highlighted pixels can be seen overlaid (*right*) on a representative unstimulated cell. *C*, increase in the number of pixels having higher B values (*magenta pixels*, *B* > 1.5) that represent the oligomeric species or clusters of eGFP-EGFR without and with EGF stimulation (*left*), and in cells treated with IQGAP1 siRNA (*right*). *D*, Western blot showing the expression of EGFR in cells both at a basal state and after EGF stimulation in the presence and absence of IQGAP1 siRNA (*left*). The Western blot intensities of EGFR are quantified after normalization with actin (*right*). *E*, Western blot showing the expression of p-EGFR in cells both at a basal state and after EGF stimulation (100 ng/ml) in the presence and absence of IQGAP1 siRNA (*left*). The Western blot intensities of p-EGFR are quantified after normalization with housekeeping gene GAPDH (*right*). *F*, the Western blot intensities of p-EGFR are ratioed with a corresponding EGFR expression after normalization with a housekeeping gene. n ≥ 10 at least two individual experiments for imaging studies. n ≥ 4 for Western blots where each n represents individual experiment. ∗*p* ≤ 0.05, ∗∗*p* < 0.01, ∗∗∗*p* < 0.001, and ∗∗∗∗*p* < 0.0001. Scrambled (non-specific) siRNA treatment is used for the control groups. Error bars denote standard deviations. Scale bar = 10 μm.

EGF stimulation of EGFR causes internalization through endocytosis to terminate the signal (12). When we stimulate HeLa cells expressing eGFP-EGFR with EGF, we see punctate cytosolic populations of EGFR after 30 min as the receptor is internalized, and this population survives if the stimulation is sustained (Fig. 2*A*). We used N&B to determine whether changes in eGFP-EGFR oligomerization are associated with clustering after long-term EGF stimulation. We find that EGF activation increases EGFR oligomerization both on or close to the plasma membrane and in the cytosol in accord with its activation on the plasma membrane and subsequent internalization both at physiological (33, 34, 35, 36, 37) and ultrahigh, non-physiological doses (Fig. 2, *B* and *C*, *left*).

To determine whether IQGAP1 contributes to EGFR oligomerization, we down-regulated IQGAP1 expression by ∼90% as estimated by western blotting and compared to a commercially available scrambled siRNA control (38). Knockdown of IQGAP1 prevents EGF-induced EGFR oligomerization (Fig. 2*C*, *right panel*), indicating that IQGAP1 promotes increased EGFR oligomerization. Interestingly, we find that down-regulating IQGAP increases the protein levels of EGFR. Since IQGAP may decrease protein levels, this finding supports the idea that IQGAP specifically promotes EGFR oligomerization and that the increased oligomerization is not simply due to increased concentration. Note that changes in EGFR phosphorylation are not dependent on IQGAP levels (Figs. 2*D* and S2 *in detail*). Even though IQGAP1 knockdown increases EGFR expression, it results in a weaker EGF-mediated increase of EGFR activity (Figs. 2*E* and S3 *in detail*). Specifically, when normalized to EGFR levels, we find that downregulation of IQGAP1 decreases EGF-mediated EGFR phosphorylation despite the overall increase in EGFR expression (Fig. 2*F*).

**Figure 3:**
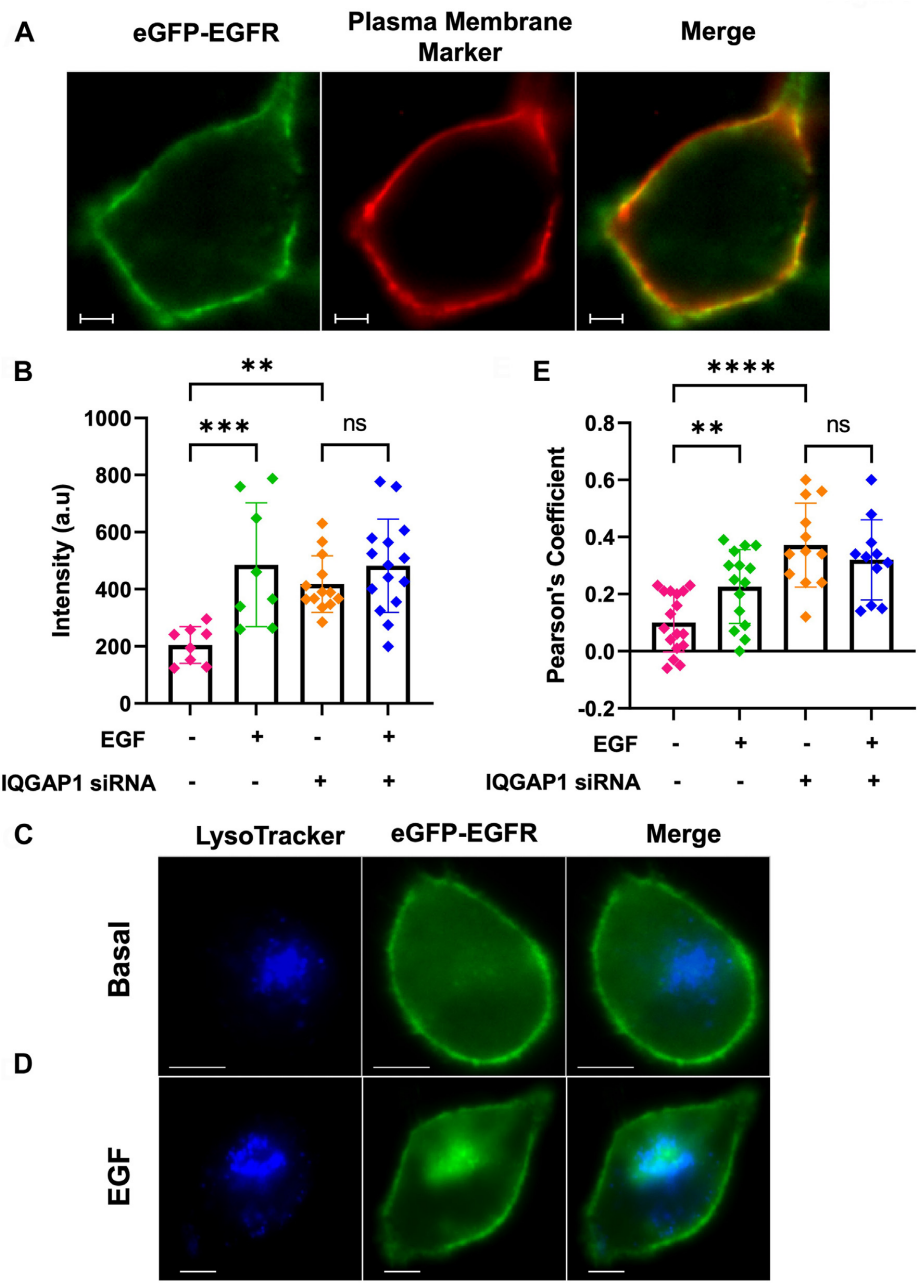
**IQGAP1 enhances EGFR localization to the plasma membrane and decreases localization to the lysosomes.***A*, a representative image showing the colocalization between eGFP-EGFR and plasma membrane marker (*red*) in HeLa cells. *B*, eGFP intensity quantified along the plasma membrane of HeLa cells expressing eGFP-EGFR before and after stimulation with EGF. *C*, a representative image showing colocalization between eGFP-EGFR and LysoTracker in HeLa cells. *D*, a representative image of a similar cell after stimulation with EGF. *E*, the colocalization between eGFP-EGFR and lysosomes was quantified using Pearson’s coefficient. n ≥ 10 at least two individual experiments, ∗∗∗*p* < 0.001. Scrambled (non-specific) siRNA treatment is used for the control groups. Error bars denote standard deviation. Scale bar = 10 μm.

IQGAP1 can regulate EGFR by modulating EGFR’s localization. To see whether this is the case, we assessed the localization of eGFP-EGFR in unstimulated HeLa cells by visualizing its colocalization with a red plasma membrane marker (Fig. 3*A*). We find that EGF stimulation increases the intensity of eGFP-EGFR on the plasma membrane (Fig. 3*B*), but this effect is eliminated when IQGAP1 is downregulated, indicating that IQGAP1 may enhance membrane localization of EGFR.

IQGAP1 is known to localize in the cytoplasm (5) with various organelles such as endosomes (39) and lysosomes (40) that are involved in the recycling and degradation of receptors as a part of receptor trafficking. We find that the cytoplasmic populations of eGFP-EGFR co-localize with lysotracker (an indicator of lysosomes), indicating that EGFR is being targeted to the lysosomes (Fig. 3, *C* and *D*), and EGF stimulation shifts this EGFR population to lysosomes. Importantly, knockdown of IQGAP1 results in a statistically significant (*p* < 0.0001) increase in EGFR-lysosome localization and eliminates any EGF-induced effects (Fig. 3*E*). Overall, these results indicate that IQGAP1 expression protects EGFR from lysosome degradation.

### EGFR–phosphoinositide association is enhanced by IQGAP1

IQGAP1’s binding to EGFR is known to be modulated by PI(4,5)P_2_ (41) and PI(3,4,5)P_3_ levels (42). Here, we determined whether EGFR binds PIP_3_ and whether this association is sensitive to EGF stimulation by monitoring changes in FRET (Förster resonance energy transfer) between eGFP-EGFR and the PI(3,4,5)P_3_ sensor mCherry PH-Akt1. The presence and extent of FRET can be measured by the decrease in donor lifetime that occurs when energy is transferred to an FRET acceptor. Fluorescence lifetime imaging microscopy (FLIM) allows us to measure the changes in the fluorescence lifetime in each pixel in the image allowing us to determine where in the cell image FRET occurs.

**Figure 4:**
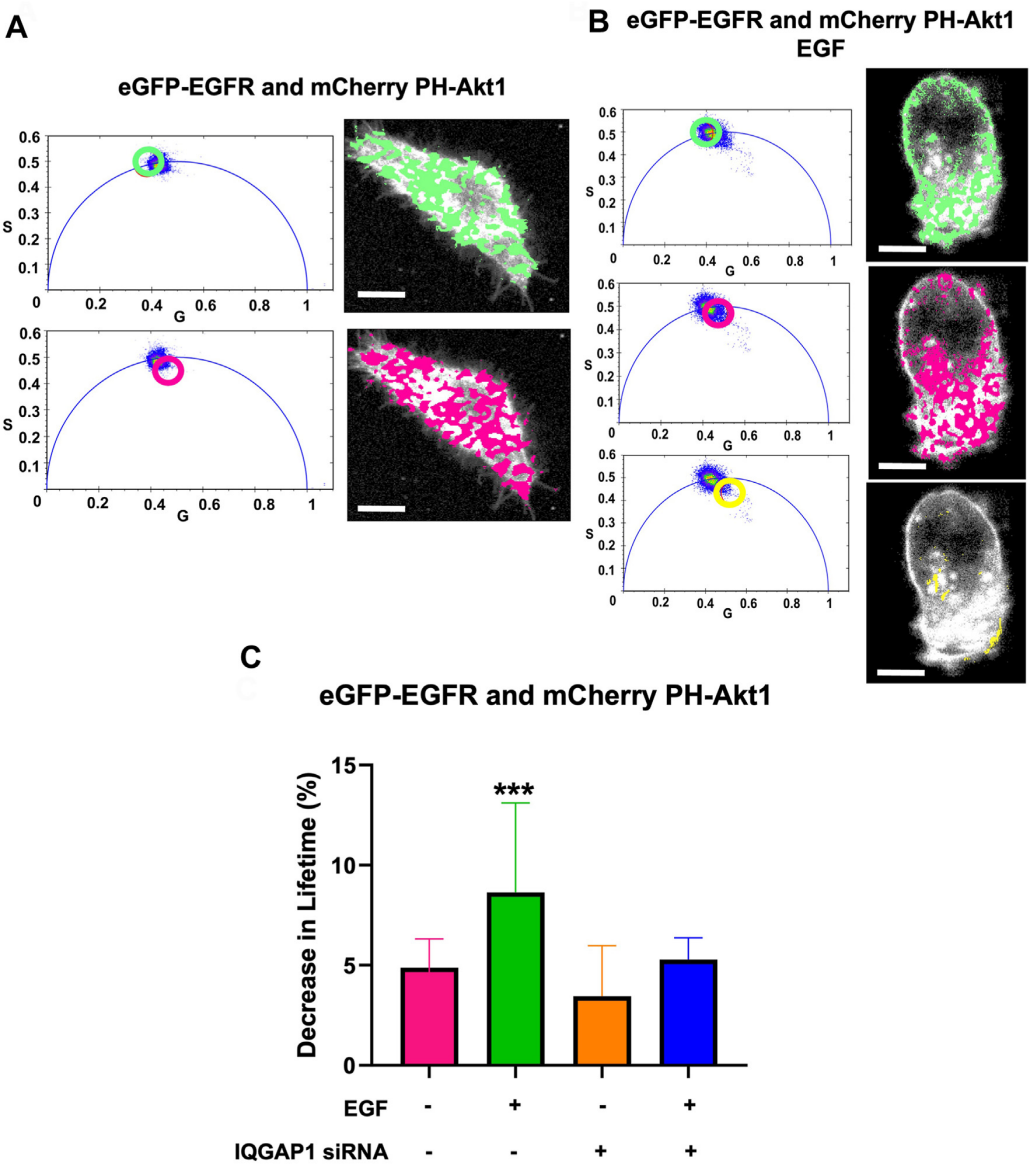
**EGFR–phosphoinositide association is enhanced by IQGAP1.***A*, images of a representative HeLa cell co-expressing eGFP-EGFR and mCherry PH-Akt1, where the pixels of distinct regions on phasor plots (*left*) are highlighted to show the distinct lifetime regions, and are represented by a *green circle* indicating higher (non-FRET) lifetimes (lifetime center = 2.55 ns) or by a *magenta circle* indicating shortened lifetimes (lifetime center = 2.00 ns). The pixels underlying these circles are false colored and overlaid on grayscale cell images (*right*). *B*, images of a representative HeLa cell co-expressing eGFP-EGFR and mCherry PH-Akt1 stimulated by EGF, where the pixels of distinct regions on phasor plots (*left*) are highlighted to show the distinct lifetime regions and are represented by *green circle* indicating higher (non-FRET) lifetimes (lifetime center = 2.55 ns) or by *magenta* or *yellow circles* indicating shortened lifetimes (*magenta* lifetime center = 2.00 ns, *yellow* lifetime center = 1.60 ns). The pixels underlying these circles are false colored and overlaid on grayscale cell images (*right*). *C*, FLIM-FRET between eGFP-EGFR and mCherry-PH Akt1 showing an increased upon EGF stimulation reducing with IQGAP1 downregulation. All eGFP lifetimes are statistically lower compared to eGFP lifetimes in cells expressing only eGFP-EGFR where n ≥ 5 measured in at least two independent experiments, ∗*p* < 0.05, ∗∗*p* < 0.01, ∗∗∗*p* < 0.001, and ∗∗∗∗*p* < 0.0001. Error bars denote standard deviation. Scale bar = 10 μm.

Additionally, we can visualize the lifetimes of each pixel of the image in phasor coordinates. In phasor plots, single populations of lifetimes are clustered on the phasor arc. Because FRET reduces the lifetime, these points appear inside the phasor arc (Fig. 4, *A* and *B*). This visualization allows us to directly assess FRET from the raw data in a model-independent way (see (43)). Choosing a particular pixel cluster on the phasor plot (shown in green, purple, and yellow circles) and overlaying the spatial distribution of these lifetimes on cell images enables the visualization of the cellular distribution of these lifetimes (Fig. 4, *A* and *B*). In FRET experiments, the non-FRET lifetime population (green) have a homogenous lifetime located on the semi-circle on a phasor plot. FRET populations are denoted by a shift inside the phasor arc (purple and yellow regions) due to the multiexponential nature of the decay caused by fluorescence lifetime decrease.

Although FRET is present in the unstimulated and stimulated case, we observe increased FRET between eGFP-EGFR and mCherry PH-Akt1 upon EGF stimulation. This increased FRET population is distributed across the whole cell including the plasma membrane (Fig. 4, *A* and *B*). Downregulating IQGAP1 eliminates this EGF-enhanced increase in the EGFR-PH-Akt1 association (Fig. 4*C*), suggesting that IQGAP1 enhances an interaction between EGFR and PI(3,4,5)P_3._

## Discussion

Scaffold proteins play key roles in enhancing or attenuating cell signals by organizing and localizing pathway proteins to promote their associations in specific cellular localization. Here, we have studied the ability of the ubiquitous scaffolding protein, IQGAP1 to mediate EGF signals in HeLa cells. By imaging live cells expressing fluorescent-tagged proteins, we were able to directly observe the impact of IQGAP1 on EGFR’s localization, aggregation, interactions with ligands, and movement to lysosomes. We note that the overexpression of fluorescent-tagged proteins may influence the impact of the behavior seen here, but our expression levels are low relative to the levels of endogenous proteins, as seen by single molecular fluorescence data.

IQGAP1 is characteristically over-expressed in various types of cancers (17) including hepatocellular (18, 19), prostate (44), glioma (21), head-and-neck (22) and breast (23). IQGAP1’s cellular localization and expression levels impact various signaling systems, and redistribution of IQGAP1 from the cytoplasm to the membrane correlates to tumor grade and poor disease outcome prognosis (21, 23, 44, 45). Here, we find that stimulation of HeLa cells with EGF increases the plasma membrane localization of IQGAP1 suggesting that the increase in cellular calcium, PIP_2_ hydrolysis, and subsequent lipid and protein phosphorylation help drive IQGAP1 membrane association. It is notable that the increase in the plasma membrane pools of IQGAP1 is accompanied by an increase in IQGAP1 homo-oligomerization correlating with the increased effective concentration on the quasi-two-dimensional membrane surface. The enhanced IQGAP1 membrane localization and oligomerization on the membrane surface in turn concentrates EGF pathway components to the membrane surface which may be associated with the increase EGF stimulation seen in cancer (13, 46). We note that our measurements cannot clearly distinguish between proteins localized on the plasma membrane and proteins incorporated in plasma membrane associated vesicles that may appear during receptor internalization or endosomal protein delivery to the membrane. Thus, enhanced membrane localization and oligomerization by IQGAP may be either direct or indirect.

We find that IQGAP1 knockdown increases EGFR expression. We also see in preliminary studies that the knockdown of IQGAP1 results in higher basal EGFR phosphorylation but a weaker EGF-mediated increase of EGFR activity. EGFR phosphorylation is a result of EGFR homodimerization caused by agonist stimulation that culminates in the activation of downstream signaling. When normalized to the increased overall levels of EGFR protein, these results show that the downregulation of IQGAP1 decreases EGF-mediated EGFR phosphorylation despite the overall increase in EGFR expression.

Our studies suggest that the cytosol-localized population of EGFR is linked to endosomes and lysosomes. Interestingly, we show that IQGAP1 expression protects EGFR against lysosomal degradation revealing a new aspect of IQGAP1’s promotion of EGFR signaling. It is possible that the increase in EGFR levels with IQGAP1 knockdown occurs in response to increased lysosomal trafficking of the receptors. We also see that IQGAP1 is localized close to the pools of PI(3,4,5)P_3_ generated by PI3K activity which is enhanced by EGF.

**Figure 5:**
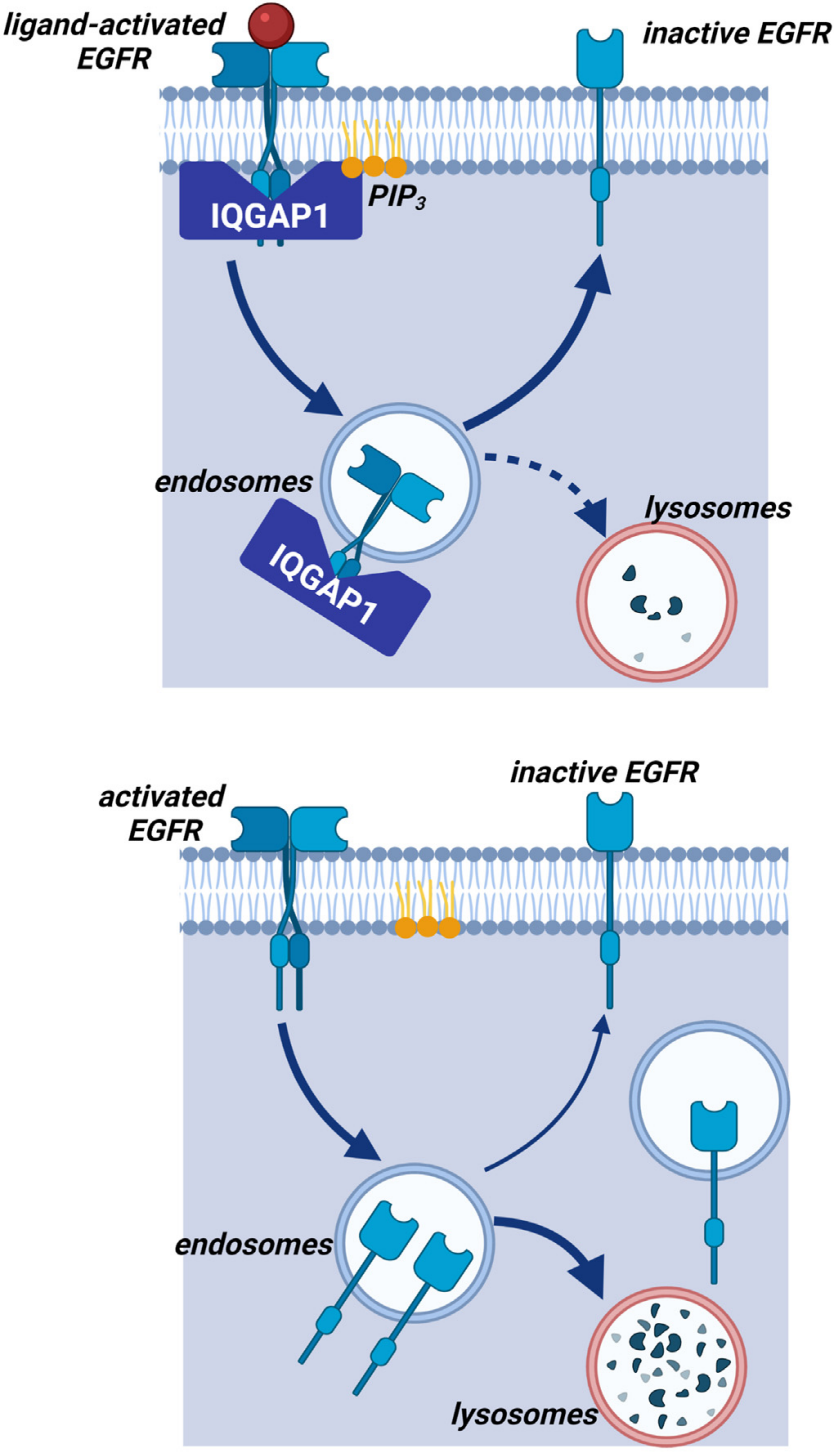
**Schematic of how IQGAP enhances EGF signaling.** A two-part schematic showing the effect of IQGAP1 on EGF-mediated stimulation of its receptor EGFR. The *top* schematic represents IQGAP1 enhancing EGF signaling by promoting the oligomerization of its receptor, EGFR upon EGF addition along with concurrent IQGAP oligomerization. The *bottom* schematic shows that in the case of downregulation of IQGAP1, there is a weakened EGFR activation that is potentially caused by the loss of IQGAP1’s protective effect on EGFR against lysosomal degradation.

In summary, results show a multi-pronged promotion of EGFR signaling by IQGAP1, especially in the context of ligand activation, as depicted in Figure 5. These mechanisms include promotion of EGFR activation, protection of EGFR from degradation, and activation of PI3K/Akt and Ras/Erk signaling pathways. Our studies further reveal the many facets of IQGAP1’s role in mediating EGF signaling, hence indicating the potential of targeting IQGAP1 for novel anti-cancer therapies that can supplement the existing anti-EGFR chemotherapy.

## Experimental procedures

### Materials

HeLa cells were obtained from the American Type Culture Collection. Untagged eGFP and mCherry plasmids were obtained from Clontech. Anti-fungal agents were added to the media and cell contamination was visually assessed throughout the studies. All other plasmids were obtained through AddGene from the following investigators: eGFP-IQGAP1 (Dr David Sacks), mCherry-PH-Akt1 (Dr Moritoshi Sato). On-Target Plus SmartPool IQGAP1-siRNA (a pool of four different siRNAs) (GE Dharmacon) was used to knockdown IQGAP1 while the scrambled siRNA was obtained from Ambion (Thermo Fisher Scientific). Lysosomal (Lysotracker) and membrane (CellMask Orange Plasma Membrane having an excitation-emission spectra of 554/567 nm) markers were also obtained from Thermo Fisher.

### Western blotting

HeLa cells were lysed with 500 μl of buffer containing 150 mM NaCl, 20 mM HEPES, 2 mM MgCl_2_, 5 mM 2-mercaptoethanol, 1% NP40, 1 mM phenylmethylsulfonyl fluoride, and cOmplete protease inhibitor cocktail tablet (Roche/Millipore Sigma). The lysed cells were boiled in sample buffer at 95 °C for 3 min and were analyzed using SDS-PAGE and Western blotting.

### Cell culture

HeLa cells were incubated in high-glucose DMEM (Thermo Fisher Scientific) supplemented with 5% fetal bovine serum (Atlanta Biologicals, Flowery Branch, GA, USA) at 37 °C with 5% CO_2_. Transfection of plasmids and small interfering RNA (siRNA) was performed using Lipofectamine 3000 (Thermo Fisher Scientific) in the antibiotic-free medium as per the manufacturer’s instructions. For treatment with agonist, cells were cultured in a serum-free high-glucose DMEM for 2 h, following which they were supplemented with 100 ng/ml EGF, unless otherwise noted, in accord with previous studies using HeLa cells (33, 34, 35, 36, 37) and incubated for at least 1 h. Experiments were performed on different days using completely different cell cultures as replicates. Cells are plated on MatTek chambers (MatTek) for imaging purposes.

### Live cell imaging

Live cell images were taken on a Zeiss LSM 510 confocal microscope while the cells were incubated in a custom-built chamber at 37 °C and 5% CO_2_.

### Immunofluorescence

For immunofluorescence studies, cells were fixed using 3.7% formaldehyde and permeabilized with 0.2% nonyl phenoxypolyethoxylethanol (NP40) in phosphate-buffered saline (PBS) for 5 min and then blocked in PBS containing 1% BSA for 1 h. Cells were then incubated with the primary antibody diluted to 1:1000 for 1.5 h at 37 °C, followed by incubation with Alexa-labeled secondary antibody for 0.5 h at the same temperature. Cells were washed with Tris buffered saline (TBS) solution after each incubation.

Images were obtained with a Nikon (Minato) Eclipse Ti inverted microscope with a TIRF Illuminator and a Nikon 60× CFI Apo TIRF oil objective. For excitation sources, coherent 488 nm, 561 nm, and 647 nm sapphire lasers were used. Images were captured with an Andor3iXon CCD camera. The images were analyzed using Nikon software and/or ImageJ (47) (National Institutes of Health).

### Image analysis

The live-cell fluorescence and immunofluorescence images were first analyzed using the manufacturer’s software (Nikon or Zeiss as appropriate). The fluorescence intensities are quantified using ImageJ (47) (National Institutes of Health). For region-specific fluorescence, the plasma membrane region or cellular regions are outlined using membrane-bound protein or membrane marker fluorescence or using brightfield imaging as a reference point. Co-localization analysis was performed using ImageJ. To avoid bias, the analysis and the imaging were performed by different individuals where possible.

### Number and brightness (N&B) analysis

The Number and Brightness (N&B) analysis is a powerful tool that has been used previously to quantify graphically the aggregation state of diffusing proteins in living cells (32, 48, 49, 50). N&B analysis can determine the number (*N*) of diffusing particles within a given focal area and the intrinsic brightness (*B*) of each particle represented by pixels in an image and provides a map of brightness for every pixel. While we used the *B*-value as a measure of aggregation and oligomerization, it does not increase linearly with increased oligomerization. The mathematical equations that describe the apparent brightness *B* for every pixel are:

(1) $$B=\frac{\sigma^{2}-\sigma_{0}^{2}}{<I>-offset}\text{,}$$

where *I* denotes the intensity of the signal, *σ*^2^ is the variance of the signal, $\sigma_{0}^{2}$ is the readout noise variance of the detection electronics and offset is the detector offset. Based on the control measurements, we chose to use the percentage of pixels with *B* values above 1.5 to quantify the oligomeric aggregates in the cells. The analysis has been described in more detail (38, 49, 50).

N&B studies were performed, by acquiring images of live cells plated on MatTek chambers (MatTek) using a 2-photon MaiTai laser (Spectra-Physics), a Nikon inverted confocal microscope in an ISS Alba System. The Images were analyzed using ISS VistaVision and SimFCS 4 software packages.

### Fluorescence lifetime imaging microscopy (FLIM)

FLIM is a method that allows to measure physical interactions as quantified by Förster resonance energy transfer (FRET) between labeled proteins in cells. FRET reduces the fluorescence lifetime of a donor molecule due to the transfer of energy to an acceptor. Because of its steep distance dependence in the low nanometer range, FRET transfer requires a direct physical interaction between the proteins to which the probes are attached. The methodology has been discussed previously (38).

In our experiments, we used green fluorescent protein donors (such as eGFP, enhanced Green Fluorescent Protein) with red fluorescent proteins such as mCherry and dsRed acting as the FRET acceptors. FLIM was performed by acquiring images of live cells plated on MatTek chambers using a 2-photon MaiTai laser (Spectra-Physics) (excitation 850 nm at 80 MHz) and a Nikon inverted confocal microscope in an ISS Alba System. The Images were analyzed using ISS VistaVision and ImageJ software packages. Atto 425 fluorescent dye (t = 3.61 ns) was used to calibrate the sample lifetimes.

### Fluorescence correlation spectroscopy (FCS)

FCS measurements were performed on cells expressing eGFP using a 2-photon MaiTai laser (Spectra-Physics) (excitation 930 nm at 80 MHz) and a Nikon inverted confocal microscope in an ISS Alba System The beam waist, *ω*_0_, and focal volume were calibrated with various concentrations of Alexa 488 dye (*D* = 425 μm^2^/s). Measurements of each cell were taken over 30 s and repeated more than four times. For each cell, the traces were averaged to obtain the final autocorrelation function to be used for fitting. The power of the excitation laser was adjusted such that there was sufficient signal-to-noise ratio and minimal photo bleaching.

(2) $$G\left(\tau \right)=1+\frac{1}{N}\left\{{\left(1+\frac{\tau }{\tau D}\right)}^{-1}{\left(1+\frac{\tau }{S^{2}\tau D}\right)}^{-\frac{1}{2}}\right\}\text{,}$$

where τ is the correlation time, τD is the average time a particle spends in the confocal volume, *N* is the average number of molecules in the confocal volume, and *S* is the structural parameter. *S* was set to 100 (quasi-infinite) for two-dimensional diffusion. The diffusion coefficient, D, is calculated from the τD of a molecule using the Einstein relation for diffusion: *r*^2^ = 4D × τD, where r is the radius of the observation volume. Curve fitting was performed using a 3D gaussian fitting model using ISS VistaVision software. Here, we report a single overall diffusion coefficient because fitting curve to multiple diffusions did not lower the fitting error.

### Statistics

All data were analyzed using Sigmaplot (SysStat) or Prism. For comparison between two groups, we used Student’s *t* test substituted by Mann-Whitney’s rank sum test if the data was not normally distributed. For multiple comparisons, we used ANOVA supplemented by Fisher’s least significant difference method (in case of normal distribution) or Dunn’s test in case of non-normal distribution. Any difference between the two groups was considered statistically significant only with a *p*-value below 0.05.

## Data availability

All primary and analyzed data are available upon request.

## Supporting information

This article contains supporting information.

## Conflict of interest

The authors declare that they have no conflicts of interest with the contents of this article.
