# Supplementary material for: The scaffolding protein IQGAP1 enhances EGFR signaling by promoting oligomerization and preventing degradation
Source: J Biol Chem. 2024 Sep 30;300(11):107844. doi: 10.1016/j.jbc.2024.107844 (PMC11555339; doi:10.1016/j.jbc.2024.107844)
Supplement: Supporting Information [file mmc1.docx]

IQGAP1 enhances EGFR signaling by Promoting Oligomerization and Preventing Degradation

V. Siddartha Yerramilli^1^, Guanyu Lin, Jessica L. Reisinger, Rachel M. Hemmerlin, Samantha K. Lindberg^,^ Karin Plante, Alonzo H. Ross, Arne Gericke and Suzanne Scarlata^*^

**Supporting Information**

Fig S1: IQGAP1 forms membrane complexes in response to EGF

Fig S2: Western Blots (anti-EGFR) shown in Figure 2D

Fig S3: Western Blots (anti-pEGFR) shown in Figure 2E

**Figure S1:** **IQGAP1 forms membrane complexes in response to EGF**:


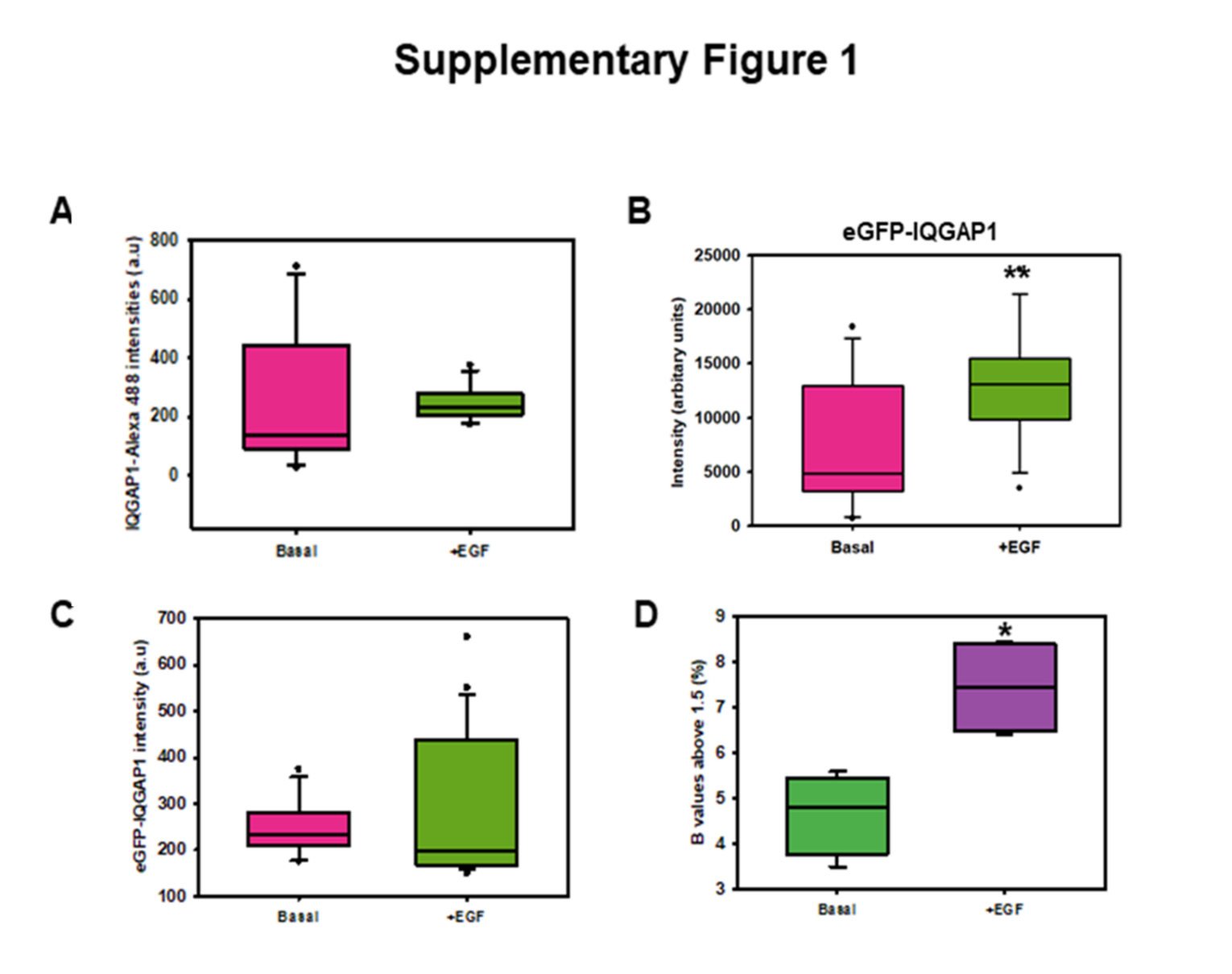
A) There is no statistically significant change in the fluorescence of endogenous IQGAP1 (stained with anti-IQGAP1 antibody) in fixed HeLa cells (n ≥10, p=0.606) in response to EGF stimulation (n ≥10, 100 ng/ml)

B) eGFP intensity is quantified along the plasma membrane on images of HeLa cells expressing eGFP-IQGAP1 before and after stimulation with 100 ng/ml EGF, and was seen to significantly increase along the plasma membrane in response to EGF

C) There is no statistically significant change in eGFP-IQGAP1 cell intensities in live HeLa cells (p=0.110) in response to EGF stimulation (100 ng/ml)

D) N&B results of cells expressing eGFP-IQGAP1 are plotted using a Brightness vs Intensity plot, where individual pixels of the HepG2 cell images expressing eGFP-IQGAP1 are separately quantified based on the B value (above and below 1.5) There is a statistically significant increase in the number of pixels having higher B values (B >1. 5) that represent the oligomeric species or clusters of eGFP-IQGAP1 after the cells are stimulated with EGF (100 ng/ml) compared to basal levels

n ≥10* = p ≤0.05, **=p<0.01, ***=p<-0.001, ****=p<0.0001. Error bars denote standard deviation.

**Figure S2: Western Blots (anti-EGFR) shown in Figure 2D**


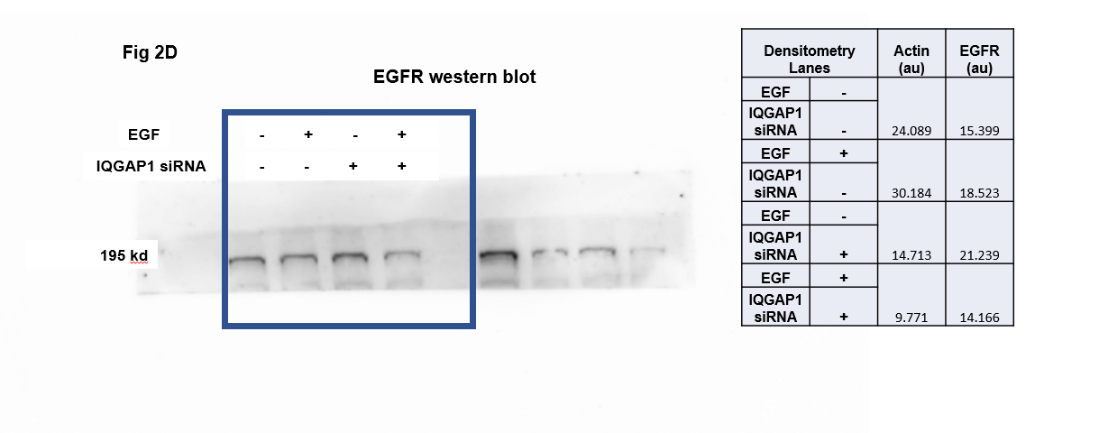


**195 kDa**

**A**

***250 kDa***

***180 kDa***

**B**


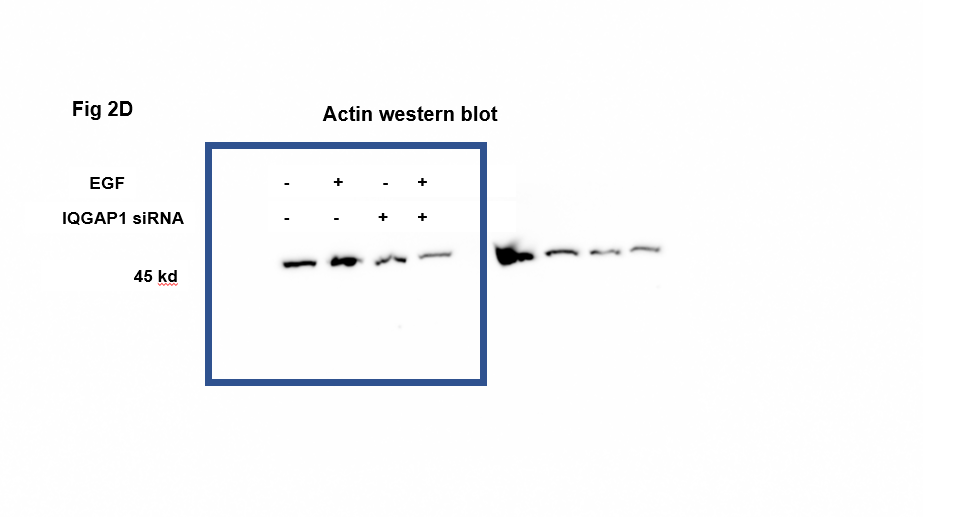


**45 kDa**


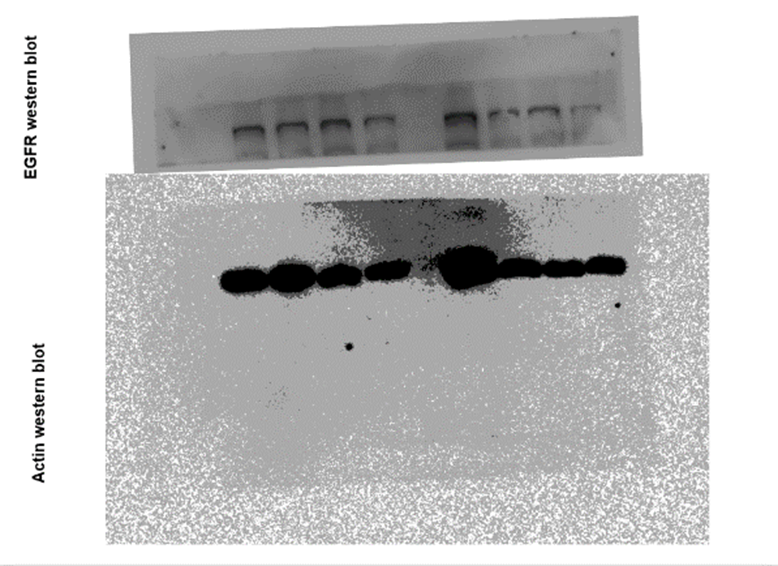


**C**

Figure S2: **Western Blots shown in Figure 2D**

1. Full western blot shown in Fig 2D stained with anti-EGFR antibody and its densitometry.
2. Full western blot shown in Fig 2D stained with anti-actin antibody and its densitometry.
3. A saturated image showing the original western blot that was cut into two for blotting separate antibodies.

**Figure S3: Western Blots (anti-pEGFR) shown in Figure 2E**


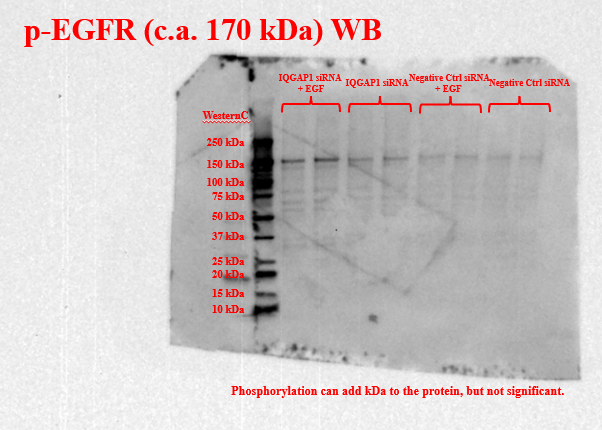
**A**


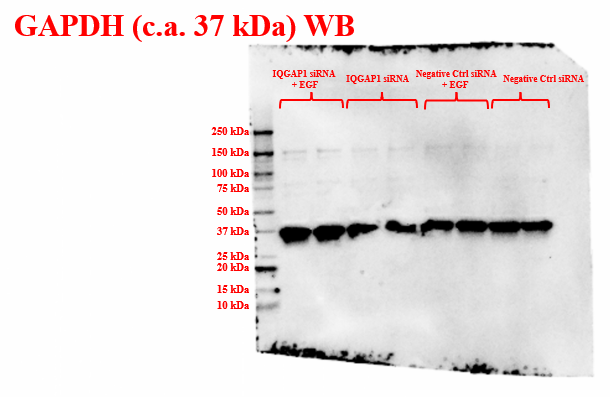
**B**

Figure S3: **Western Blots shown in Figure 2E**

1. Full western blot shown in Fig 2D stained with anti-pEGFR antibody.
2. Full western blot shown in Fig 2D stained with anti-GAPDH antibody.
